# Supplementary material for: Assessment of deep learning segmentation for real-time free-breathing cardiac magnetic resonance imaging at rest and under exercise stress
Source: Sci Rep. 2024 Feb 14;14:3754. doi: 10.1038/s41598-024-54164-z (PMC10866998; doi:10.1038/s41598-024-54164-z)
Supplement: Supplementary file 1 — Supplementary Information. [file 41598_2024_54164_MOESM1_ESM.pdf]

Supplementary Information

Assessment of Deep Learning Segmentation for Real-Time Free-Breathing Cardiac Magnetic Resonance Imaging at Rest and Under Exercise Stress

Martin Schilling*,[1] Christina Unterberg-Buchwald*,[1,2,3] Joachim Lotz,[1] and Martin Uecker†,[1,2,4]

[1] *Universitätsmedizin Göttingen, Institute for Diagnostic and Interventional Radiology, Göttingen, Germany*
[2] *German Centre for Cardiovascular Research (DZHK), Partner Site Göttingen, Göttingen, Germany*
[3] *Universitätsmedizin Göttingen, Clinic of Cardiology and Pneumology, Göttingen, Germany*
[4] *Graz University of Technology, Institute of Biomedical Imaging, Graz, Austria*

* *these authors contributed equally to this work*
† *corresponding author's e-mail: uecker@tugraz.at*

Supplementary Table S1. Acquisition parameters for cine and real-time CMR
All volunteers were measured with the same protocol. Cine and real-time CMR were acquired using a bSSFP sequence. Real-time CMR used iterative image reconstruction with NLINV.

	cine	real-time
Sequence type	Cartesian	radial
Number of images per slice	25	120-150
Slice thickness [mm]	6	6
Field of View [mm^2]	340 x 276.25	256 x 256
Matrix size [pixel]	256 x 208	160 x 160
Spatial resolution [mm^2]	1.33 x 1.33	1.6 x 1.6
Flip angle [degree]	49	23
Echo time [ms]	1.53	1.28
Temporal resolution [ms]	30.1	33

Supplementary Table S2. Comparison of 2D nnU-Net on single and stacked images
The segmentation accuracy of the 2D nnU-Net inference is compared for the application on single and stacked images. Although the 2D nnU-Net is applied on single images, the inference with the 2D nnU-Net features test time augmentation and normalization, which take all input data into account. To determine the optimal form of application for our task, we applied the 2D nnU-Net on single images and stacks of multiple images for cine CMR and real-time free-breathing CMR at rest, under exercise stress, and under maximal exercise stress. The table features the mean Dice's coefficients for the left ventricular endocard (LV), the left ventricular myocardium (MYO), and the right ventricle (RV) for all volunteers. For RT max stress, only data of 12 volunteers were analyzed, because in three cases the image quality was too poor to create reasonable reference contours. Only images in the end-diastolic and end-systolic phase were segmented. In our case, segmentation benefitted from test time augmentation and normalization of single images. The differences in segmentation accuracy between the application on single images and stacks of images increase for more challenging segmentation tasks. For cine CMR, images with the same cardiac phase are stacked along the slice dimension. For real-time CMR, images of a time series in a single slice are stacked.

	**LV**		**MYO**		**RV**	
n=15	single	stack	single	stack	single	stack
cine	0.952	0.951	0.907	0.907	0.904	0.898
RT rest	0.943	0.912	0.885	0.859	0.896	0.849
RT stress	0.921	0.833	0.850	0.775	0.826	0.674
RT maxstress (n=12)	0.909	0.788	0.826	0.748	0.788	0.598

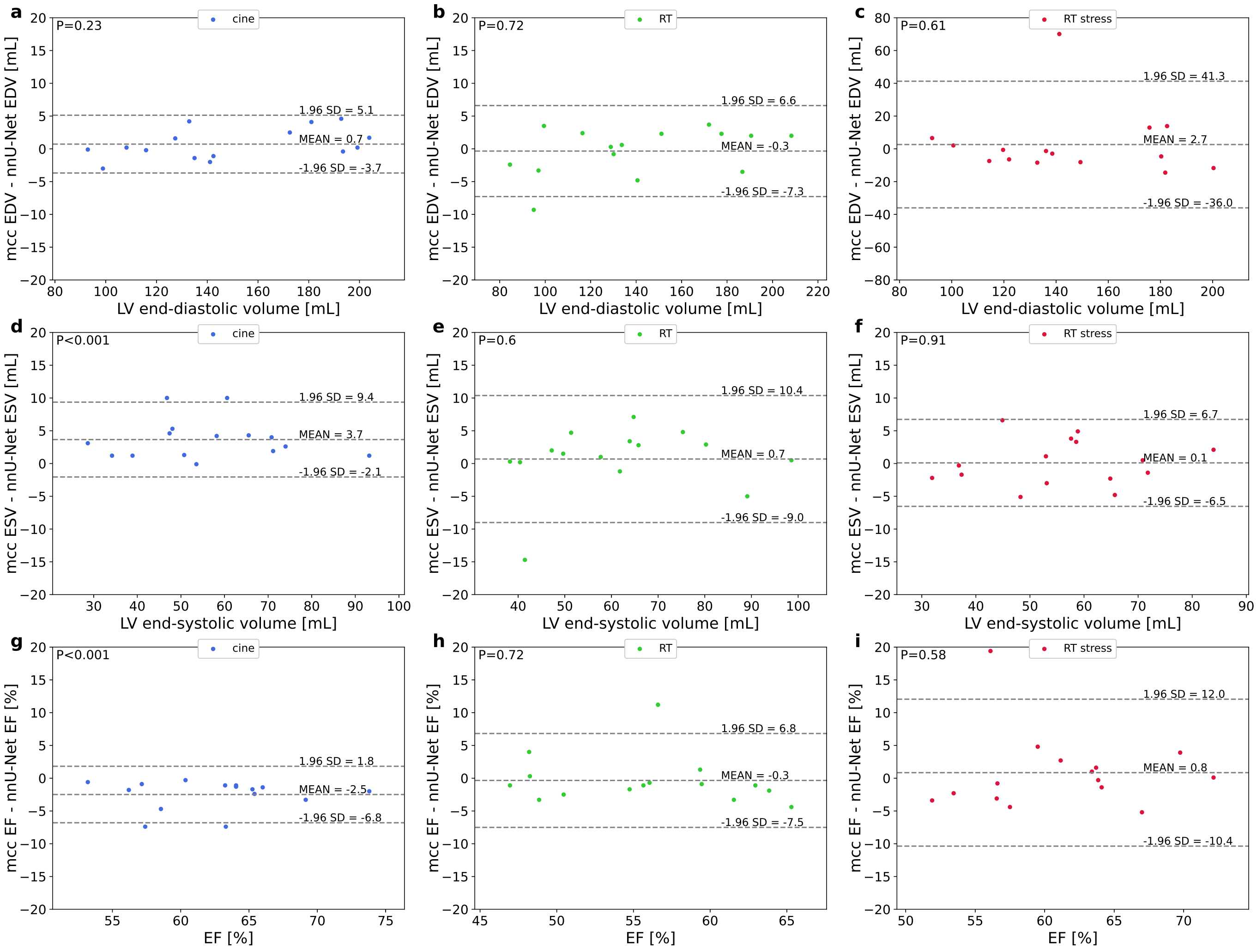

Supplementary Figure S1. Bland-Altman plots for cardiac function parameters of nnU-Net
The cardiac function parameters of (**a-c**) the left ventricular end-diastolic volume (EDV), (**d-f**) the left ventricular end-systolic volume (ESV), and (**g-i**) the left ventricular ejection fraction (EF) derived from the nnU-Net segmentation and the manually corrected contours (mcc) are compared with each other in Bland-Altman plots. The parameters are compared for cine CMR (**a**, **d**, **g**), real-time free-breathing CMR at rest (**b**, **e**, **h**), and under exercise stress (**c**, **f**, **i**). P values of a paired two-sample t-test are shown in the top left corner of each plot.

Supplementary Table S3. Comparison of 2D nnU-Net, 3D nnU-Net, and their ensemble
The segmentation accuracy of the 2D nnU-Net, the 3D nnU-Net, and their ensemble is evaluated for cine CMR of all volunteers. Only slices containing the left ventricle have been stacked, similar to the ACDC dataset. Only images in the end-diastolic and end-systolic phase were segmented. The table features the mean Dice's coefficients for the left ventricular endocard (LV), the left ventricular myocardium (MYO), and the right ventricle (RV) for all volunteers.

n=15	LV	MYO	RV
2D nnU-Net	0.951	0.907	0.898
3D nnU-Net	0.791	0.589	0.292
ensemble nnU-Net	0.926	0.852	0.833

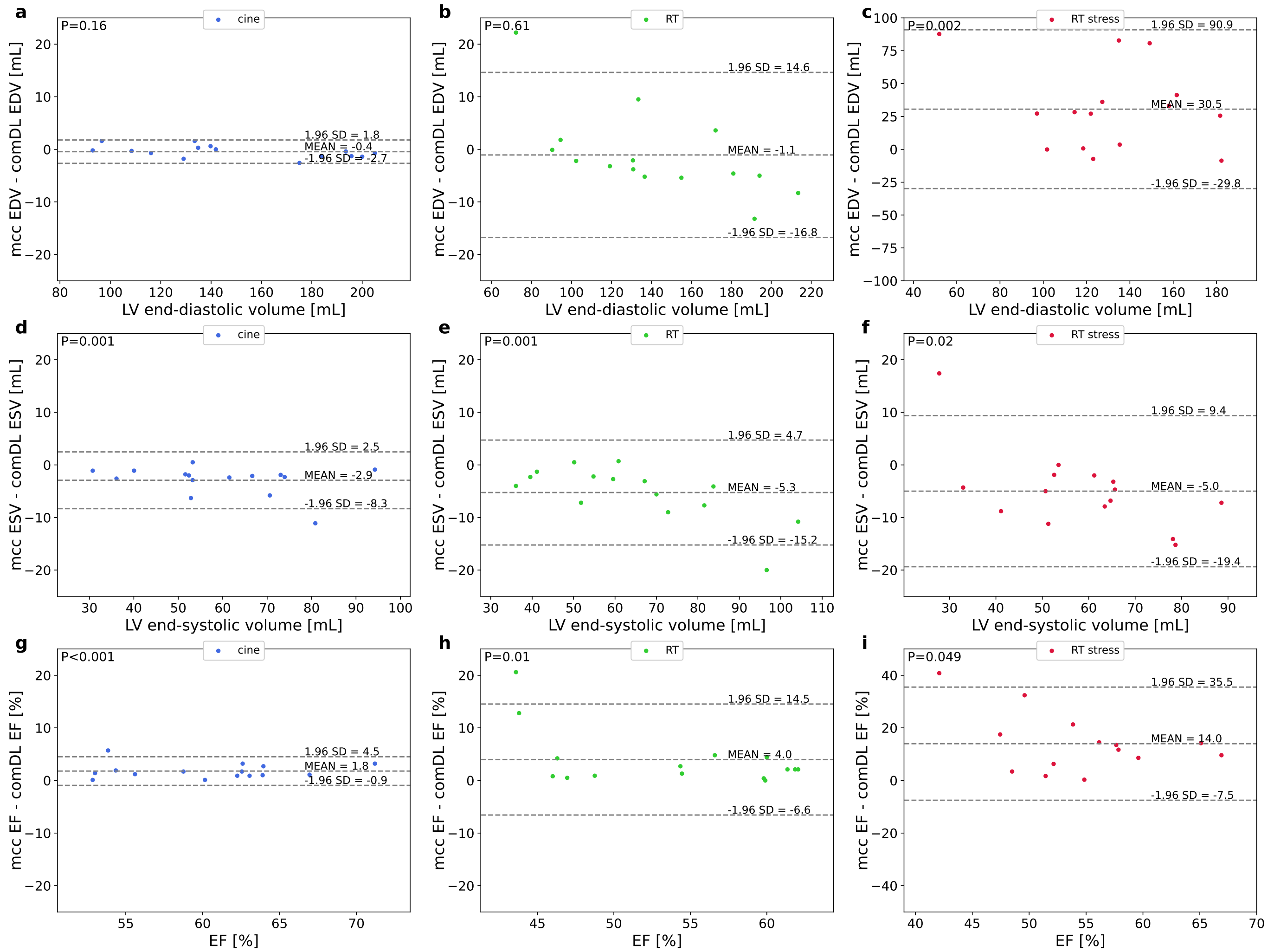

Supplementary Figure S2. Bland-Altman plots for cardiac function parameters of comDL The cardiac function parameters of (**a-c**) the left ventricular end-diastolic volume (EDV), (**d-f**) the left ventricular end-systolic volume (ESV), and (**g-i**) the left ventricular ejection fraction (EF) derived from the comDL segmentation and the manually corrected contours (mcc) are compared with each other in Bland-Altman plots. The parameters are compared for cine CMR (**a**, **d**, **g**), real-time free-breathing CMR at rest (**b**, **e**, **h**), and under exercise stress (**c**, **f**, **i**). One data point for EF at RT stress (**i**) has been omitted as the EF value derived from comDL contours was negative. *P* values of a paired two-sample t-test are shown in the top left corner of each plot.

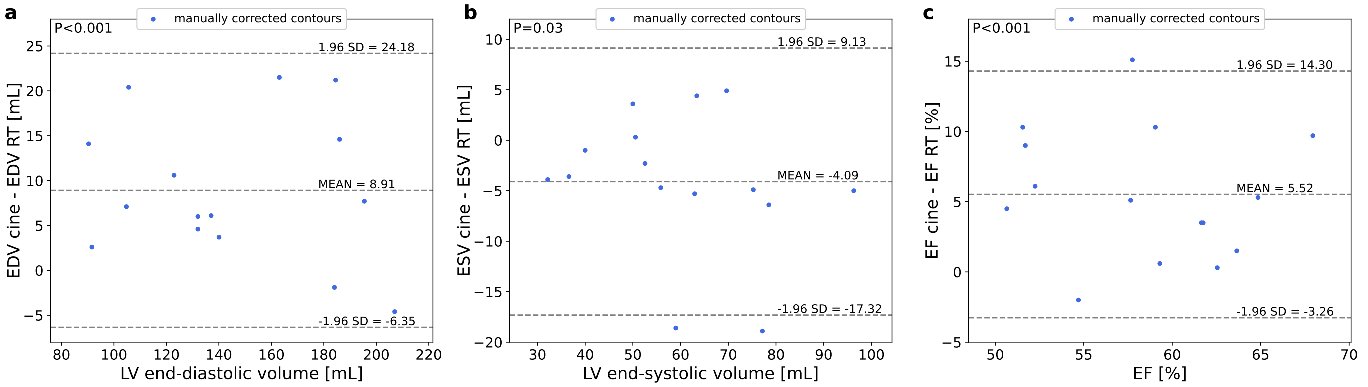

Supplementary Figure S3. Comparison between cine and real-time CMR cardiac function parameters The cardiac function parameters of (**a**) the left ventricular end-diastolic volume (EDV), (**b**) the left ventricular end-systolic volume (ESV), and (**c**) the left ventricular ejection fraction (EF) derived from manually corrected contours for cine and real-time free-breathing CMR at rest are compared with each other in Bland-Altman plots. P values of a paired two-sample t-test are shown in the top left corner of each plot.

Supplementary Table S4. Calculated heart rates for real-time measurements Calculated heart rates in beats per minute for real-time free-breathing CMR at rest (RT rest), during exercise stress (RT stress), and maximal exercise stress (RT maxstress). For the calculation of heart rates, we used the three central slices of all slices between the base and apex. Heart rates were calculated based on the time span between consecutive end-diastolic phases. The mean and standard deviation (in parenthesis) is given for each volunteer. Missing entries were not segmented due to poor image quality.

n=15	RT rest [bpm]	RT stress [bpm]	RT maxstress [bpm]
vol 01	66 (2)	113 (4)	150 (8)
vol 02	67 (5)	111 (7)	140 (8)
vol 03	63 (3)	114 (4)	122 (14)
vol 04	55 (2)	109 (7)	134 (5)
vol 05	53 (2)	117 (6)	—
vol 06	68 (1)	109 (6)	140 (8)
vol 07	73 (3)	119 (7)	147 (8)
vol 08	77 (3)	115 (7)	140 (10)
vol 09	75 (3)	120 (5)	164 (13)
vol 10	68 (3)	108 (4)	155 (13)
vol 11	65 (4)	116 (6)	—
vol 12	76 (3)	114 (6)	162 (11)
vol 13	74 (3)	108 (10)	—
vol 14	64 (3)	111 (6)	121 (6)
vol 15	57 (1)	107 (6)	122 (10)

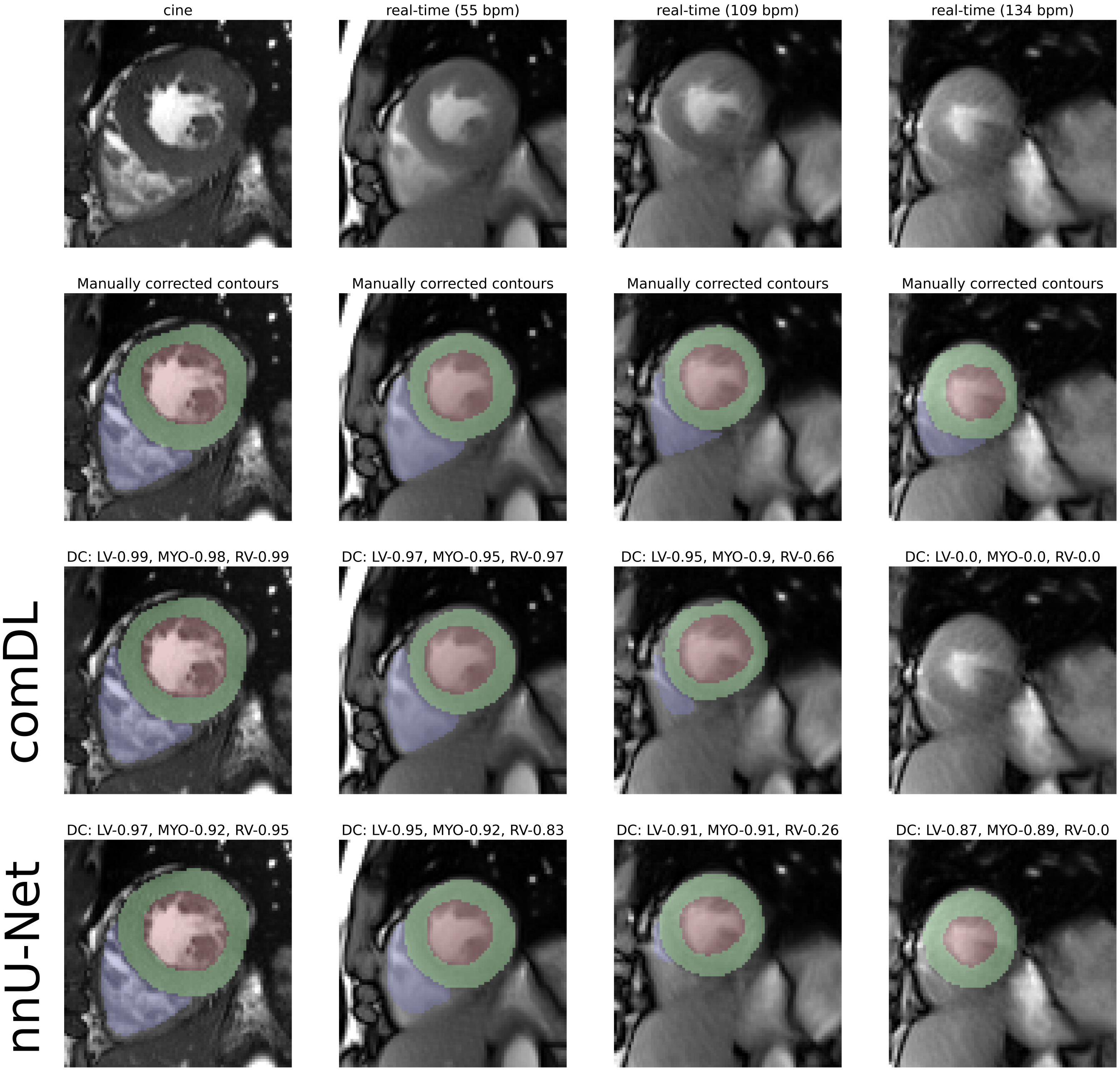

Supplementary Figure S4. Segmentation failure of right ventricle segmentation outlier of nnU-Net Mid-ventricular slices in ES phase of a volunteer for cine and real-time free-breathing at different heart rates (first row) with corresponding manually corrected (second row), comDL (third row), and nnU-Net segmentation (fourth row). Accuracy of segmentation is measured with Dice's coefficient (DC). DC for left ventricular endocard (LV), left ventricular myocardium (MYO), and right ventricle (RV) are given for each segmentation. The images for real-time stress (109 bpm) and real-time maxstress (134 bpm) are segmentation failures of the two outliers of RV segmentation of the nnU-Net. For these images, the transition between RV and the neighboring liver tissue seems to be too smooth for a successful segmentation. The comDL segmentation fails completely for real-time maxstress, while the nnU-Net segments LV and MYO with good accuracy.

Supplementary Table S5. Cardiac function parameters for cine and real-time CMR
The absolute values of the cardiac function parameters of the left ventricular end-diastolic volume (EDV), the left ventricular end-systolic volume (ESV), and the left ventricular ejection fraction (EF) are given for each volunteer for cine CMR and real-time free-breathing CMR at rest (RT) and during exercise stress (RT stress).

(**a**) EDV [mL]

n=15	cine	RT	RT stress
vol 01	128.2	117.6	119.4
vol 02	141.9	138.2	145.3
vol 03	92.9	90.3	95.8
vol 04	193.3	178.7	176.3
vol 05	204.7	209.3	189.5
vol 06	135.0	129.0	137.1
vol 07	140.1	134.0	135.5
vol 08	173.8	152.3	178.0
vol 09	115.8	95.4	118.8
vol 10	97.4	83.3	108.3
vol 11	199.3	191.6	194.5
vol 12	108.3	101.2	101.7
vol 13	183.1	185.0	182.3
vol 14	134.3	129.7	128.6
vol 15	195.1	173.9	174.6

(**b**) ESV [mL]

n=15	cine	RT	RT stress
vol 01	51.8	48.2	59.5
vol 02	65.6	61.2	63.7
vol 03	30.2	34.1	36.5
vol 04	72.1	67.2	60.2
vol 05	72.8	77.7	71.1
vol 06	60.3	65.6	61.3
vol 07	51.4	53.7	48.2
vol 08	75.3	81.7	85.0
vol 09	50.7	50.4	53.5
vol 10	34.8	38.4	33.1
vol 11	93.8	98.8	71.1
vol 12	39.5	40.5	36.7
vol 13	49.7	68.3	51.6
vol 14	53.5	58.2	45.7
vol 15	67.7	86.6	63.3

(**c**) EF [%]

n=15	cine	RT	RT stress
vol 01	59.6	59.0	50.2
vol 02	53.7	55.7	56.2
vol 03	67.5	62.2	61.9
vol 04	62.7	62.4	65.8
vol 05	64.4	62.9	62.5
vol 06	55.3	49.2	55.3
vol 07	63.4	59.9	64.4
vol 08	56.7	46.4	52.3
vol 09	56.2	47.2	55.0
vol 10	64.2	53.9	69.4
vol 11	52.9	48.4	63.4
vol 12	63.5	60.0	63.9
vol 13	72.8	63.1	71.7
vol 14	60.2	55.1	64.5
vol 15	65.3	50.2	63.7
